# To: *Ralstonia pickettii* bacteremia in hemodialysis patients: a report of two cases

**DOI:** 10.5935/0103-507X.20160082

**Published:** 2016

**Authors:** Vitorino Modesto dos Santos

**Affiliations:** Hospital das Forças Armadas - Brasília (DF), Brazil; Universidade Católica de Brasília - Brasília (DF), Brazil.

**To the Editor,**

I recently read the interesting report by Tejera *et al*. about *Ralstonia pickettii*, a gram-negative germ formerly of the *Burkholderia* group that infected two patients on hemodialyisis.^(1)^ The first was a 65-year-old man with a chronic kidney disease and native arteriovenous fistula who, and was managed in the intensive care unit (ICU) because of development of a septic shock.^(1)^ The transesophageal echocardiogram did not show vegetation, but cultures of blood and of dialysis fluid cultures revealed *R. pickettii*, which was controlled by with meropenem for two weeks.^(1)^ The second was a 45-year-old hemodiyalytic man with chronic rejection of a kidney transplant who was admitted to the ICU because of fever, chills and hypotension.^(1)^ The transthoracic echocardiogram showed a mitral vegetation. Moreover, the cultures of blood and of dialysis fluid cultures revealed *R. pickettii*, which was treated with piperacillin-tazobactam for three weeks.^(1)^

As discussed by the authors, this emerging opportunistic pathogen found in domestic and hospital water may cause severe bacteremia infections and septic shock related to health care.^(1)^ It is worth noting, that the gram-negative agents in venous catheters are a source of endocarditis.^(1,2)^ Persistent neutrophilic leukocytosis and spiked fever despite empirical antibiotic therapy should raise the hypothesis of endocarditis, and an echocardiogram can confirm the vegetation.^(1)^ The authors called attention to major issues about gram-negative endocarditis that can occur in healthcare-associated infections, especially in patients with catheters for hemodialysis.^(1)^

Gram-positive bacteria are the main cause of infectious endocarditis in hospitalized patients, but uncommon agents, such as *Klebsiella, Salmonella* and *Burkholderia*, have been described.^(2)^ In this setting, comments should be made about a Brazilian woman with a renal transplant and a diagnosis of endocarditis by *Burkholderia cepacia* associated with an intracardiac foreign body. This foreign body was a fragment of a peripherally inserted central catheter positioned 16 years earlier during her treatment in the ICU for a postpartum episode of septicemia and circulatory shock.^(2)^ The cardiac foreign body remained undetected until it was infected by the circulating bacteria, probably because of corticosteroids and muromonabe-Cd3 (OKT3) utilization after her renal transplant.^(2)^ The catheter fragment detected by imaging studies was removed by a cardiotomy procedure. The 40-year-old woman was treated with a combination of trimethoprim and sulfamethoxazole, considered the first option for her treatment in accordance with the antibiotic sensitivity test.^(2)^

The authors emphasized the role of protocol for in treating renal transplant patients, with a special investigation of possible infective foci, even in the absence of fever or overt signals of infection; unapparent cardiac foci can evolve in hemodialytic patients and those with end-stage renal disease.^(2)^ As a major risk factor for infectious endocarditis in this particular group of individuals, the possibility of infection at the site of the dialysis catheter should be evaluated during daily care. Peripherally inserted central catheters often utilized in ICUs may be associated with fragmentation and infection, which constitute a source of endocarditis.^(2)^ The aforementioned cases of gram-negative endocarditis involving chronic renal patients illustrate the following two practical points: evident clinical features enhance the suspicion of health workers as to the possibility of endocarditis^(1)^ and routine protocol for renal transplant patients can yield an initially unsuspected diagnosis.^(2)^

Vitorino Modesto dos Santos

Hospital das Forças Armadas - Brasília (DF), Brazil; Universidade Católica de Brasília - Brasília (DF), Brazil.

## References

[1] Tejera D, Limongi G, Bertullo M, Cancela M (2016). Ralstonia pickettii bacteremia in hemodialysis patients: a report
of two cases. Rev Bras Ter Intensiva.

[2] Falcão Pedrosa Costa A, Castelo Branco Cavalcanti F, Modesto dos Santos V (2014). Endocarditis due to Burkholderia cepacia and an intracardiac
foreign body in a renal transplant patient. Rev Port Cardiol.

